# Supplementary material for: A novel theoretical framework for simultaneous measurement of excitatory and inhibitory conductances
Source: PLoS Comput Biol. 2021 Dec 28;17(12):e1009725. doi: 10.1371/journal.pcbi.1009725 (PMC8746761; doi:10.1371/journal.pcbi.1009725)
Supplement: Attachment — Submitted filename: PLOSCompBio_resubmission_point-by-point001.pdf [file pcbi.1009725.s002.pdf]

Dear Editors and reviewers, *Sep 17, 2021*

We would like to thank you for the objective and fair assessment of our work. One of the major concerns raised by you was the lack of validation with experimental data. We would like to mention, however, that a couple of months ago we started a new collaboration, with Prof. Alexander Binshtok from the Hebrew University aimed to validate our method for simultaneous measurement of excitatory and inhibitory inputs in a single cell and a single trial. Our recordings of neurons in brain slices, although showing promising results, are not consistent enough to be included in this current work. In this experimental work we were able to see that some of our predictions from the model were indeed validated. For example, the increasing impedance at high frequency during evoked synaptic response. Hence, we strongly believe that we will be able to validate our method in the near future. However, it is well possible that some modification in the instrumentation, perhaps even at the level of the intracellular amplifier, will be required to fully and rutinly implement the method. In short, it is clear that validation of our method in real recordings is beyond the scope of this study , which is focused on laying the computational foundation for such measurement. There are numerous examples from physics, in which theories for measurements were established long before technologies matured enough to allow full implementation of the method. We hope, therefore, that this matter will be fairly considered by the reviewers and the editors.

Reviewer #1: Comments to the Authors

In this paper, authors provided a novel method to simultaneously extract excitatory and inhibitory synaptic conductances of a single neuron from the recorded membrane potential at a single trial. The method is analytical and looks very interesting. It uses two high frequency sinusoidal components (as injected current) and solves the general equation of subthreshold membrane potential of a single neuron for the total conductance (Gs) and the electrode resistor (Rs). From these items, and also estimating the cell's capacitor, the proposed method infers Ge and Gi as excitatory and inhibitory synaptic conductances, respectively. To me this method is very novel. However, I vote to reject just because this method has the potential to be improved and validated with experimental data. Having said that, I should highlight that the authors provided very nice simulations to demonstrate the accuracy of the method. As well, all the points in the Discussion are appreciated. I have some major points as follow.

1. This method should be tested with experimental data

   We appreciate that the reviewer believes our analysis and simulations are sound and we agree that the method should be tested experimentally. In the past 2 months we have started a new collaboration with another group in which we will perform intracellular recordings in brain slices. These experiments prove to be difficult because of inconsistency in our results As we mention in the discussion, we believe that these issues have to do with the bandwidth at which contemporary current clamp amplifiers can reliably deliver alternating currents, an issue that was also mentioned by the second reviewer. Although some of the results we obtained recently are very promising, they are not consistent enough to be presented at this stage, before we

understand this issue. While we are carefully working through these hardware challenges, we strongly believe that our simulations and the theoretical description of the method are worth publishing as they will be critical to improve the method in the future. This may help in establishing new collaborations, perhaps by combining our method with other methods (see also the comments made by the second reviewer and our response).

2. What is the impact of observation noise on the accuracy of estimated Ge and Gi?

We thank the reviewer for suggesting that we test the impact of noise on the accuracy of our measurements. We decided to simply add a real noise, recorded using patch pipette in an in-vitro setup, to the simulated voltage. The results of this simulation are now shown in a new figure (Fig. 7). The data demonstrate that although - as expected - the measurements are a little bit noisier, it is still feasible to measure both synaptic inputs.

3. How the clean voltage is calculated? Is it filtered again?

The clean voltage is obtained by bandstop filtering around (+-15Hz) around each injected frequency (two processes in series). We now mention this clearly in the revised manuscript.

4. If I understood correctly, the idea of injecting two sufficiently high frequency injected currents was for estimating Gs and Rs (as well as C). Although I really liked this idea, I do not understand why authors do not inject more than two currents, for example 5 high frequency currents, and solve those conductances using a simple optimization technique (without any assumption or simplification).

This is an excellent point that we are still considering. We simulate two sinusoidal inputs because this case is analytically tractable and does not require any optimization. This has some advantages, for example computational efficiency. Since the primary hardware issue seems to be injection bandwidth, we are also concerned that experimental implementation could become more complicated with multiple frequencies and we also think (as we examine) that the solutions are less stable numerically. Since this was the case in our initial investigation we decided to opt for the two frequencies alone.

More explicitly, we tried 3 frequencies and towards this approach we solved the circuit system for 3 simultaneously injected frequencies (to find total G, C, Rs) and found that errors increased when adding a tiny amount of capacitance to the electrode. We believe that optimization will require making an assumption on one or more measured variables during the process of measurement. Because in our approach, we make only few and trivial assumptions (such as the reversal potentials and the point model of the cell), the solution can be applied to a wider range of conditions.

Reviewer #2: # Comments to the Authors

Authors propose a frequency-domain method to infer simultaneously the excitatory and inhibitory (summated) time-varying conductances, from a single intracellular neuronal recording. As opposed to existing methods, Authors first simulate a single-trial current-clamp stimulation in a a passive RC circuit, while recording its electrical potential. They later explore the performance of the method in another numerical simulations, employing a multicompartment biophysically realistic CA1 model cell.

I disclose that I must have seen an earlier and more primitive exposition of this idea as a 2019 bioarXiv entry. It greatly captured my interest as an experimentalist. I think that the concepts behind the method are definitely of immediate interest for the (more) specialised audience of a methods journal (e.g. Neuron, J. Neurosci. Method) - even though the method has not (yet) been demonstrated by the Authors in an experiment - and less for the audience of a Comp Neurosci journal.

We deeply appreciate the assessment of the reviewer and support. In particular we are grateful for the high quality check and constructive comments, aimed to improve the big picture of our presentation and the fine details of the text and figures.

Clearly, our goal is to implement the method in real experiments. As we disclosed in our manuscript we made attempts to use this method in real recordings (see also the answer to reviewer 1 and the general statement we wrote as opening remark in this point by point answer). As mentioned above, since we submitted the manuscript, we have started a new collaboration in which we would like to reveal the excitation and inhibition evoked by optogenetic stimulation in brain slices. Although the results are promising , they are not consistent enough across cells. As we mentioned above it is well possible that these inconsistent results may stem from the sensitivity of the recording amplifier, we may need to modify the amplifier and thus will significantly delay the ability to obtain proof of concept. We believe that without fully understanding the reasons for these inconsistent results, it will be premature to provide a proof of concept in this current manuscript. In summary, we believe that this work has the potential to facilitate additional collaborations with other experts in intracellular recordings. These experts may provide other solutions that will improve this method.

We strongly believe that our study, which is purely computational, is well placed in PLOS Comp. Bio., which publishes computational research with biological relevance.

I recommend major revisions.

Major points

1)

The presentation of the manuscript could definitely be improved. Math notation and precision in using throughout the text is not "stable" and should be revised carefully.

Especially the description of the math could be made a bit clearer, taking a bit more of presentation, discussion and analysis. Some of the steps skipped (definitely accessible for a reader with a math or engineering background) might make a naive reader lost.

We believed that we made the math description stable and clearer. We also improved the explanation for the mathematical steps in many cases. We also had the manuscript read by colleagues to make sure it is accessible. We hope that the reviewer finds these changes sufficient.

2)

I recommend an improved exposition of the hypotheses behind the use of frequency-domain method, with quasi-stationary parameters. Strictly speaking, eq. 3 is obtained from eq. 1, upon Fourier transformation, but a sort of "separation of time-scales" has been invoked, adopted, but not illustrated. While Fig. 2 is obtained keeping g_e and g_i fixed in time, eq. 3 assumes they can vary. If they do vary with time, then Fourier-transforming eq. 1 is no longer straightforward in the frequency domain.

I see of course that this does work in simulations, as a reverse method, but it still lacks of a justification and a more extensive discussion.

Circuit analysis is usually done for fixed stationary circuit components. However, as long as the sine wave input does not interfere with the values of the components (for example when the components are affected by the fluctuating voltage) there is no reason why the separation will not work. Other studies (such as those below that were mentioned by the reviewer) in which sine wave signals were used to estimate changes over time of the capacitance of neurons have made similar assumptions. The theoretical transformation from equation 1 to 3 is valid as long as the changes in voltage are not influencing the values of the components of the circuit. This is not necessarily true in real cells due to voltage dependent ion channels, and this is why we would like to keep the fluctuations of the sinusoidal voltage across the membrane as small as possible (in our simulations <5-6 mV).

3)

The choice of the sample values of the electrode resistance (30 and 50 MOhm) seems unusual at a first sight. Could it be that it includes the access resistance? This is mentioned nowhere and it seems anyway not typical in "good" (in vitro) recordings. Perhaps do Authors refer to access resistance in vivo? Please explain and give some references to the literature.

*We thank the reviewer for this comment. Indeed, this resistance includes the access resistance and it is true that these values are higher than expected for typical 'good' in-vitro recordings. Yet, eventually our primary goal is to apply this approach for in-vivo recordings, in which access resistance is higher. Hence, testing the method when access resistance is high is crucial. We refer to this matter in the section '**The in-silico experiment'.** More importantly, our method deals with changes in access resistance as we demonstrated in Figure 6 ('**Measurements of Ge and Gi are accurate even when electrode resistance is not stable').** Such changes in access resistance are often observed in real recordings. This issue was clarified in the revised version and a reference was added.*

Does your method for estimating the capacitance have similarities with this classic 1988 paper by Neher (10.1007/BF00582306) and this 1997 extension (10.1016/S0006-3495(97)78810-6), using two sine waves ? A discussion of similarities and differences with past approaches might be useful for the curious reader.

These methods share some similarities with our method, primarily because in our study, like those mentioned above, we analyzed the impedance (or admittance) of cells when sinusoidal signals are applied to the cells. We note, however, that as far as we know, the goal of these methods is to measure small changes in capacitance value and thus lock-in amplifiers are used under voltage clamp recordings. Since we are not deeply familiar with the capacitance measurement methods we prefer to only briefly refer to these methods in the manuscript. The more advanced double sine wave method that was mentioned by the reviewer allows estimation of more variables at the same time while making fewer assumptions in order to solve the equivalent circuit. However, since in the essence of our method, measurements must be conducted under current clamp in order to reveal excitatory and inhibitory conductances we believe that except for using the theory of circuit analysis and sinusoidal signals, our method is fundamentally different. Nevertheless, we have added a short sentence into the Discussion as follows: "We note that our method shares the basic approach for the analysis of point-neurons using the theory of frequency analysis of electrical circuits as was for capacitance measurements (Neher and Marty 1982; Lindau and Neher 1988). "

Could the approach of Badel et al. (10.1007/s00422-008-0259-4) be of any relevance for the capacitance estimation (see their Fig. 1c)?

Thank you very much for mentioning this paper. Indeed, the method described in the paper of Badel et al. looks elegant and promising. It requires injection of noise followed by variance

analysis. It is well possible that we will apply this method in case that we find that our capacitance measurement method, when applied in future experiments, will provide poor estimation of the cell's capacitance. However, the estimation of the capacitance, when using the approach of Badel (et al. 2008), will require additional trials in which noise will be injected, and thus more experimental time. In our approach, however, the capacitance is estimated from the same trial in which excitation and inhibition are measured. We may contact Badel (et al. 2008) in the future if we find that our approach is a limiting factor. We refer to this alternative method for capacitance measurement in the section "Measurement of the cell total conductance " as follow:

"Other methods for such estimation are also available, such as using short pulse *<u>(Durand et al. 1983; Lampl and Yarom 1997)</u>* *and variance analysis of the response to injection of noise* *<u>(Badel et al. 2008)</u>. "*

4)

Can you comment on the opportunities/obstables represented by online parametric (concentional bridge balance and capacitance neutralisation circuitry) and (offline) non-parametric (as in the Active Electrode Compensation of Brette and colleagues - see 10.1016/j.neuron.2008.06.021)?

Particularly the latter method should (theoretically) capture the impulse response of pipette and amplifier. That might ameliorate your implementation problems.

We thank the reviewer for suggesting to us this interesting method for active compensation by Brette (et al.) from the group of Alain Destexhe. This important method indeed allows to subtract the electrode contribution to the recorded voltage by computing the electrode Kernel and then using it for compensation during further recordings. However, as far as we understood, this method was mainly developed for dynamic-clamp, which is not part of our method and is not planned for the physiological recordings. Moreover, we are not interested in subtracting the electrode resistance or compensating for the capacitance during the recordings, as these values, as we show, greatly affect the measurements and are captured well by our approach. In our preliminary recordings we were able to obtain promising results for some cells (as mentioned above) so perhaps this method of Brette and colleagues will be required. At this stage we are trying to improve our experimental methods, first by recording from more cells and finding which parameters are those that seem to limit the method and in parallel we plan to improve the recordings by reducing the pipette capacitance while testing different setting values of the amplifier controller (i.e., filtering parameters and capacitance neutralization). Depending on these efforts, we may contact Alain and hopefully receive more insights on his technique.

5)

I would make it more explcit that you are no longer considering any off-sets in the membrane potential and that all the quantities are referred to the "effective" resting membrane potential.

However this "effective" resting potential is, strictly speaking, not constant as it is equal to:

$$(g_l V_l + g_E(t) V_e + g_I(t) V_i) / (g_l + g_E(t) + g_I(t))$$

Can you clarify if and why these effects are negligible in your hands?

We are not sure to which section of the analysis the reviewer refers in this comment. We believe that the reviewer was referring to the steady state case, otherwise the cdv/dt term has to be included in the above equation which describes Vm(t) - unless changes in the Vm (and conductances) are slow compared to Taum. In our estimation of the conductances we took into account the capacitance. We guess that the reviewer was referring to the changes in Vm during sinusoidal current injection in which the membrane deviates from its mean potential. In our simulations the amplitude of this current was set to generate small fluctuations in Vm so the membrane doesn't deviate by much from its resting value. This is mainly because we expect that in physiological recordings large fluctuations of membrane potential are likely to activate voltage dependent conductances. We refer to these issues already in the original submission.

6)

An expansion of the discussion on "Feasibility of the technique in real recordings" is strongly suggested.

As the Authors are aware, this is the strongest selling point of the method.

We prefer to keep this section as is, without expending it further. We already obtained promising results from our in-vitro recordings, which we wish not to include in this manuscript. This is simply because we believe that a strong substantiation of the method in experiments is needed before it will be ready for publication. As mentioned above our new results are inconsistent across the recorded cells and therefore we plan to further investigate this issue.

Some aspects related to (old) patch-clamp amplifier designs criticism (see 10.1016/S0006-3495(98)74007-X and 10.1016/s0166-2236(96)40004-2) might be perhaps of use.

Thank you for the relevant and important references. Indeed, one of our concerns when implementing our method is the feedback circuits of the patch amplifiers. This feedback can greatly affect the accuracy of measurement of rapid events, such as action potentials. Rather than theoretically studying this matter, as part of the preparation for the experiments in which we are applying our method, we connected the headstage of a Multiclamp 700B to a passive circuit model composed of two resistors and a capacitor, to mimic a point neuron recorded via a pipette (neglecting the capacitance of the pipette). Luckly, using our method we were able to measure each one of these components with high accuracy (less than 5% error). Clearly, these measurements will be provided together with the experimental physiological results when our experimental study will be ready for publication. Hence, we believe that despite the limitations of the intracellular amplifiers, there are good chances that the method will work well for measurement of excitation and inhibition. In summary, we are very grateful for mentioning these articles as we may contact the authors and ask for their help in order to further improve our method.

Minor points:

1)

I suggest using (in general) "j" for the imaginary unit, to distinguish it from the current.

Thank you, we implemented this change.

I also suggest expressing eq. 3 as

$$Z(w) = R_s + 1/(G_s + j w C)$$

rather than rearranging terms to get real_part + j * imaginary_part. It will resonate immediately with people familiar with RC filters. Calculation of magnitude and phase can be anyway done quickly by the properties of the "ratios and products of complex numbers".

We added this expression to equation 3 rather than changing it.

2)

Throughout your manuscript, you considered a rather strong current injection (~1-2 nA large). I would make it clear that these are not DC stimuli, leading certainly to spiking activity. They are AC stimuli, like those employed for the analysis of subthreshold membrane resonance (see the classic work by Nelken and colleagues).

It is important to stress that the frequency is so high (compared to the membrane subthreshold cut-off frequency) that the membrane potential will be extremely attenuated and thus (almost surely) not recruiting any voltage-gated intrinsic current.

We thank the reviewer and modified the sentence in the section "The in-silico experiment" to emphasize this issue more: "Due to the low-pass filtering of the input by the passive properties of the cell when injecting high frequency sinusoidal current, the fluctuations of the voltage across the membrane itself are extremely attenuated, resulting with less than 6 mV peak to peak. Such small fluctuations are unlikely to change recruit any voltage-gated intrinsic current. "

3)

The math notation is (very) often non-uniform throughout the text, or a bit imprecise.

We made the mathematical notations more consistently throughout the manuscript.

- page 4, eq. 1 and line 8: please use (V_l, V_e, V_i) or (V^l, V^e, V^i), not both.

Both notations are needed as V^l refers to V-V_l, where V_l is the actual resting potential. This is true also for V^e and V^i.

- page 4, eq. 1, please make it clear that g_e and g_i (and g_s) are, together with V, all functions of time: make the "(t)" dependency explicit.

Done. Thanks.

- page 4, eq. 1: although conventional, the current "I" should have a positive sign on the right hand side of the equation, assuming as positive the current "entering" the cell;

It has a positive sign (note that it was included with a negative sign within the parenthesis and thus when multiplied by the minus sign outside it becomes positive).

- use, throughout the manuscript and figures, "f_1" and "f_2" (subscripted indexes) for the frequencies instead of "f1 and f2"sometimes and "F1 and F2" some other times.

Was fixed.

- use, throughout the manuscript and figures, "g_e" and "g_i" (subscript), instead of sometimes using "G_e" and "G_i".

Was fixed. All were changed to substript.

- page 7 (text and eq. 4): please use subscript for pulsations "w1", "w2", and frequencies "f1","f2", as you do it for the two components amplitude (i.e. I_1 and I_2).

Was fixed.

4)

Some text and figures contain typos.

- page 4, section title: check the extra "a";

Done

- page 8, check the spelling of "experimentalis";

Thanks. The sentence was changed and the word was removed.

- all figures: please increase your care for the "spacing" and "layout" of the subpanel titles: they are sometimes wrongly displaced and can get confusing.

We appreciate the comments on the quality of our figures. We hope that all such typos were fixed.

- Fig. 1b: check spelling of "Membrane potentia"

Fixed.

- Fig. 2, axes: please use the same symbol for frequency "f" instead of "Fr" and "Z" instead of "imped";

Done

- Fig. 2, use a single convention for indicating the units ("..., MOhm" versus "Phase(rad)")

We believe that 'rad' is a standard notation. *

- Fig. 2a,d: for consistency, use abs(Z) and phase(Z).

***done

- Fig. 2c: the black line for "data1" has not been included

Was removed

- Fig. 2c: the circuit sketch is "partly occluded" by the legend;

Fixed

- Fig. 2b,d: use "g_s" instead of "cell G", for consistency with the text;

Done. We note that the conductance we describe here is the total (g_l+g_e+g_i).

- Fig. 4c (and 6c): check spelling of "Measured Conductane";

Fixed, thanks!

- Fig. 4d,e (and 6d,e): use the same notation "g_e" and "g_i" and not another one as "E" and "I".

Done

- Fig. 4a,d (and 6a,d): check the x-axis label (removing extra space before the closed parenthesis)

Done, thanks!

Note that the caption of Fig. 3 neither refers nor illustrates panels e.

Done.

Note that the caption of Fig. 5 neither refers nor illus trates panels g,h,i,j.

Thanks! legend was revised.

Note that the caption of Fig. 6 contains a typo (i.e. "lowpassong").

Fixed.

5)

- Fig. 2b and d are difficult to grasp quickly;

We made a few changes in the caption and we hope that these panels can be grasped more quickly.

- Fig. 3f,g,h,i contain almost no information, at the choosen level of representation; consider replacing them by a block diagram, with what is input and what is the filtering/analysis operation.

We appreciate this suggestion. However, we will keep the same format for the figures as we worry that it will create too much cluttering. The order of the operations we used are clearly at the order of the panels so we feel that the method is well presented.

6) eq. 6: can you use "arctan()" instead of tan^-1 ? It would improve the clarity.

Done

7) Can you explain why you had to use Hilbert and could not use FFT ?

We guess that the question is related to equation 7. As far as we know this is the best way to get the phase of a sinusoidal signal and it worked perfectly well in these simulations.

Reviewer #3: Neurons' activity is by far determined by the interplay between the activity excitatory and inhibitory synapses on their membrane. The effect of an active synapse is to temporarily change the membrane conductance and selectively permit the current of specific ions in or out of the cell. It is thus essential to measure synaptic inputs in neurons to understand how they transform their input into an output. However, previous methods could only allow the measurement of either the excitatory or inhibitory conductance at a time. These methods were helpful in studying the response to a repeating sensory input such that the measurement could be switched from measuring excitation to inhibition. But this could not reveal their co-variation. Neither could it resolve their co-activity when there was no specific stimulus to trigger upon.

This manuscript introduces a novel protocol and analysis method to resolve the excitatory and inhibitory conductances impinging on a neuron simultaneously in a single trial. The study cleverly harnesses the resistance of the measuring electrode (which is often considered a problem/bug). It shows that the combined response of the neuron-electrode system to

sinusoidal current injections can be used to resolve the excitatory and inhibitory conductances simultaneously. This is a significant improvement on any existing method and was actually considered an unsolvable problem until now.

The manuscript is very methodological, starting with the simplest case and gradually adds a various level of complexity, solving most of the relevant cases, including variations in the electrode access resistance, electrode capacitance, and synaptic inputs located on a dendritic tree.

I strongly support the publication of the manuscript. I have no major comments on the manuscript. All of my comments relate to clarification of the text. I feel that the paper should take a more careful approach towards the notations. Some of the variables mentioned appear in different equations with different meanings, and this is difficult to keep track of.

We thank the reviewer for the support and for finding the study suitable for publication.

General comments:

1. The text on page 8 is unclear. It goes between general statements and specific examples and, in general, is not very methodic. Also, the derivation of equation 8 is not clear at all.

We are sorry for not explaining this section well enough. We hope that the changes we made help to clarify this section. We also explain the derivation of equation 8 better than before. This equation describes the trigonometric relationship between the real and imaginary parts of equation 5 using the arctan function.

2. In addition, the issue of capacitance compensation is not mentioned at this stage of the text (appears later), while clearly, this is a significant factor as the capacitance of the pipette is much larger than that of the cell. It would be wise to mention here that there is a challenge, and it will be treated later (such that the reader does not think it is ignored).

We agree that the capacitance of the pipette is potentially very problematic. This is why we made significant effort in solving this problem and included our partial solution in the manuscript. Indeed we changed the section "Measurement of the cell total conductance " and mentioned that we also can compensate for the pipette capacitance as shown later in Figure 7.

3. If I understand correctly, equation 5 basically represents the fact that the decay of the impedance is linear with w*C on the log-log plot, as seen in the plot of figure 2a. However, when the electrode is present, this linearity breaks down, as the authors demonstrate on 2c. So is the approximation in eq.5 really justified?

*In fact the opposite is true: when the electrode is present the relationship between total impedance and cell conductance becomes more linear as shown in Figure 2d. The important feature that justified eq. 5 is demonstrated in Figure 2d for the phase as function of cell conductance. These curves (shown for different frequencies) demonstrate that the phase is almost constant (almost horizontal lines). This is due to the presence of the electrode (as seen when comparing these curves to those in Figure 2b). The (almost constant) phase in these plots is determined by the electrode resistance and the capacitance of the cell and thus justifying equation 5. In other words, the approximation of equation 5 is explained in this section with examples and is based on the fact that the frequency of the sinusoidal current is high enough so the admittance of the ohmic component (i.e., g) is lower than that of the capacitance component (w*c). We also better explain now that the estimation of cell's capacitance is done by averaging for a time window for which no stimulation is provided and thus excitatory and inhibitory inputs are absent. Calculation of the cell's capacitance during this time is more accurate due to the lower values of g.*

4. The choice of the two fundamental frequencies 210Hz and 315Hz seems somewhat arbitrary. Clearly, if they are too similar there is a separation problem and if one is too high or the other too low there will be interference with the capacitance and resistance. Is there some optimization process that can be suggested to pick the "best" frequencies based on the cell capacitance and the electrode resistance?

We thank the reviewer for this comment. It is true that these frequencies were selected in a somewhat arbitrary way. Theoretically, if no noise or stray capacitance exist, any two frequencies will enable to measure the conductances of the cell. However, we cannot think about a particular optimization process that can be suggested to pick the best frequencies. The two frequencies were indeed decided based on: 1) Enough separation which improved the SNR (as expected from the reduction of the slope of the curves as frequency increases in Figure 2d). 2) The upper frequency cannot be too high, due to a reduction in the SNR when noise is present as well as interference with the feedback circuit and stray capacitance. 3) They should be as high as possible to maximize the temporal resolution of the measurement. Our decision was based on various simulations. Clearly, in the simulations themselves, we can take any two frequencies that we wish as long as these simulations are noise free and no stray capacitances exist. However, to make the simulation realistic we limited the frequency of the 2nd signal to 400 Hz as we expected that stray capacitances barely affect the response below this upper limit. To be on the safe side we reduced the frequency of the 2nd signal to about 300Hz. Recently, we found that our intuition was indeed correct: we connected to our amplifier passive RC components, mimicking the cell and the electrode, and saw that we can measure the values of these components when the frequency of the sinusoidal components is at the range we chose in this theoretical work. However, as we have explained in the general section addressed to all the reviewers, we prefer not to include any experimental results (including these measurements of passive RC circuit) in this manuscript.

5. Equations 11 & 12:

a. The notations of Equations 11 and 12 are not fully clear. The equations use:

i. “c” - which is the estimated capacitance (but the estimation is not used in the symbol)

c is indeed the estimated capacitance and we refer to it now just before the equations (without the notation so the equation is not overloaded.

ii. “Rs” – not clear which Rs is that, the real one, the estimated one from the first procedure (i.e., eq. 6?)

We now change the notations in equation 11&12 so it is clear that Rs is the estimated one from equation 11.

b. So, for example, which is the Rs that appears in equation 12 – is it Rs from equation 11 or is it Rs from equation 6.

As mentioned above, the Rs in equation 12 is the estimated one from equation 11.

c. In addition, in the text right after the equation it refers to Rs_{est2} and G_{est2} in equations 11 and 12 which do not really appear there. These notations should be revised.

These were typos and they were corrected (the subscript ‘2’ was a typo). There is only one Rs and one Rest. Thanks!

d. I might be wrong but if you use delta_w for (w1-w2) it seems that eq. 11 could be simplified.

This is true, but we prefer to keep it as is. It is also what we got directly from Mathematica and thus would like to keep it this way if someone wants to reproduce it.

e. Do the authors have an explanation why G_{est} is only dependent on z1 and w1 and not on z2 and w2? If so, it would be nice to have it in the text following eq. 12.

Excellent comment. It is actually true that if we take equation 12 and replace z1 by z2 and w1 by w2 we get the same result. We added a note for it in the text.

6. Capacitance compensation: Again, notations are not confusing. The authors define Y=1/Z and then in equation 13 they define Y1 as a different quantity which is no 1/Z so when in eq. 15 they use 1/z1 and Y1 as two different quantities it turns out to be very confusing. This is amplified in the discussion around eq. 17 and 18 where again there is a use in Rs which is used in the definition of Y but also in eq. 11 & 12 (see above).

We apologize for the confusion that was made by our notations. Equations 13-15 are theoretical and are used to conclude that Cp can be estimated as in equation 16. We changed the text and notations and hope that clarity was improved.

7. Dendritic input – eq. 19 comes as a surprise with no real justification. It also feels somewhat circular because the estimation of g_s depends on g_l. It would be nice if the authors explain how did they come up with this equation and how do they actually use it.

In fact we arrived at this equation empirically by changing the leak conductance manually before calculating the synaptic conductances. We noted that we need to lower g_l during synaptic input in order to prevent errors in the measurements. Importantly, this equation affects the g_l during the calculation of evoked excitation and inhibition of the realistic cell, and therefore it has no effect when the evoked conductances are near zero. Our intuition is that this function compensates for the changes in the electrotonic length of the cell due to synaptic input. During evoked synaptic activity impinging on proximal regions to the soma, less current escapes to the dendrites and thus the effective g_l should be smaller in the soma. When the synaptic input is small the new G_l approaches the measured g_l before the compensation was made. Our empirical function compensates for these changes. Since total SYNAPTIC g is almost zero before stimulation (we take the difference between total G and the leak conductance (g_s) and thus this calculation of E and I g's is not affected. Hence, this function affects only the measurement of evoked excitation and inhibition. In summary, we agree that this function is far from being trivial and it took us a long time to formulate it and see that it actually works well.

8. Dendritic inputs – It is well known that the conductance of distal dendritic input cannot be resolved precisely due to space clamp issues. It would be interesting to discuss here the "effective" conductance of the distal inputs. To some extent while the algorithm might not be able to restore the original conductance in the dendrite it might give a good estimate of what this conductance looks like from the soma.

We would like to thank the reviewer for this comment. Indeed due to space clamp issues the measured conductance of distal dendritic inputs are poorly estimated by our method. This is true also for the voltage clamp measurements (shown in Figure 9&10 (8&9 in the original submission). We note however, that our method is less accurate when compared to voltage clamp measurements when inputs are located at large distances. Regarding the conductance and how it looks from the soma, we are not aware of a better ground truth than multi-sweep voltage clamp. For proximal inputs however, our method provides a better estimate of the synaptic conductance compared to voltage clamp.

9. In that context consider adding citations to:

a. Häusser M, Roth A. Estimating the time course of the excitatory synaptic conductance in neocortical pyramidal cells using a novel voltage jump method. Journal of Neuroscience. 1997 Oct 15;17(20):7606-25.

b. Rall W, Segev I. Space-clamp problems when voltage clamping branched neurons with intracellular microelectrodes. In Voltage and patch clamping with microelectrodes 1985 (pp. 191-215). Springer, New York, NY.

Citations to these papers were added. Thanks!

Minor comments:

The writing could be improved. For example on pp. 3 "Hence, as these methods provide only an average picture and thus fail to capture the instantaneous and trial-by-trial based insight in the relations between excitation and inhibition."

Figure 1 – there is no legend on the figure showing which color is which.

Fixed by adding to the caption the colors.

The pass from page 5 to 6 is not obvious. It would be better to show how you are going to use the impedance in theory and only then add the pipette resistance. Right now the text seems to answer a question that wasn't even asked at that point.

We added language to the manuscript to make clear that we add the pipette resistance to our impedance analysis from the beginning. Adding the pipette resistance to the analysis makes sense to us at this point, because it is an unavoidable part of the experimental setup. Therefore, we would not want to do detailed simulations with impedance analysis, before adding the pipette resistance.

Legend of figure 2b is sloppy.

We apologise for the poor presentation of the panel and did our best to improve it.

Pp6,7 – it would be good to show on a separate panel the intersection point as a function of impedance to support the claim.

The fact that the impedance-frequency curves for different conductances intersect is an interesting observation but not a major factor in our analysis. We therefore do not think it requires a separate panel.

P8: Typo: "experimentalis"

Fixed as this sentence was changed.

P10: "(Figs. 3f to i, due to screen resolution are as patches of colors)" – looks like there is a word missing: "are displayed as patches…"?

Thank you. We added the word displayed.

Legend of figure 5 could be improved.

We carefully revised the legend and hope it is now acceptable.

Fig 8. It would be better to reverse the order of the labels (i.e., True g_e, measured g_e, VC_g_e)

Thank you. We implemented this change.
