# Supplementary material for: A novel theoretical framework for simultaneous measurement of excitatory and inhibitory conductances
Source: PLoS Comput Biol. 2021 Dec 28;17(12):e1009725. doi: 10.1371/journal.pcbi.1009725 (PMC8746761; doi:10.1371/journal.pcbi.1009725)
Supplement: Attachment — Submitted filename: Comments.docx [file pcbi.1009725.s001.docx]

**Comments**:

In this paper, authors provided a novel method to simultaneously extract excitatory and inhibitory synaptic conductances of a single neuron from the recorded membrane potential at a single trial. The method is analytical and looks very interesting. It uses two high frequency sinusoidal

Components (as injected current) and solves the general equation of subthreshold membrane potential of a single neuron for the total conductance (Gs) and the electrode resistor (Rs). From these items, and also estimating the cell’s capacitor, the proposed method infers Ge and Gi as excitatory and inhibitory synaptic conductances, respectively. To me this method is very novel. However, I vote to reject just because this method has the potential to be improved and validated with experimental data. Having said that, I should highlight that the authors provided very nice simulations to demonstrate the accuracy of the method. As well, all the points in the Discussion are appreciated. I have some major points as follow.

1) This method should be tested with experimental data
2) What is the impact of observation noise on the accuracy of estimated Ge and Gi?
3) How the clean voltage is calculated? Is it filtered again?
4) If I understood correctly, the idea of injecting two sufficiently high frequency injected currents was for estimating Gs and Rs (as well as C). Although I really liked this idea, I do not understand why authors do not inject more than two currents, for example 5 high frequency currents, and solve those conductances using a simple optimization technique (without any assumption or simplification).
