# Supplementary material for: A novel theoretical framework for simultaneous measurement of excitatory and inhibitory conductances
Source: PLoS Comput Biol. 2021 Dec 28;17(12):e1009725. doi: 10.1371/journal.pcbi.1009725 (PMC8746761; doi:10.1371/journal.pcbi.1009725)
Supplement: Attachment — Submitted filename: Letter to Editor point by point response_Nov30 - Copy.docx [file pcbi.1009725.s003.docx]

Dear Editors,	Nov 30, 2021

We would like to thank you for considering our study for publication. Our response to Milad’s comments appear below. Briefly, we think that it is too early to add any experimental results to this paper as we do not feel comfortable with publishing these results as they stand now.

We have modified the paper, mostly focusing on presentation issues, such as improving graphics and made slight changes in the grammar and equations. We would like to note that equations (8) and (19) were incorrectly written in the paper,although were fully correct in the Matlab code. We hope that our changes improved the presentation of the paper and we again thank you for handling our paper.

Reviewer #1: Thanks for your responses. All of my previous major and minor points - except that related to the experimental validation - were addressed. As well, the authors improved the quality of the paper significantly.

I can agree with the statement that " There are

numerous examples from physics, in which theories for measurements were established long

before technologies matured enough to allow full implementation of the method".

However, I want to share this idea with the authors that some detailed discussions related to your recent experience with experimental data (through your new collaboration) can be very useful for the community. It would be great if authors add a "Case Study" in the manuscript and discuss your recent results. Capitalizing on these points in Discussion (I believe you made most of them in your current manuscript) can be significantly helpful for other researchers using/trying your method.

As I am not aware of practical things with respect to this collaboration and the extent to which you can share some data and figures, I leave this idea to authors to check its possibility.

We would like to thank Milad for his insightful review. We deeply appreciate it.

Regarding the case study, we think that although promising, it will be premature to release with this paper partial results from our experimental work. Such preliminary data can lead to more confusion than to a constructive methodological description of the method. We also think that it will take many more months to tackle all the difficulties we mentioned in the paper. In case that we will not be able to solve the technical issues we see in the experimental work, we will clearly provide preprint publication as a “Case Study”.

Reviewer #2: Authors have satisfactorily addressed my concerns. I thank them for their effort and I hope we might even be able to collaborate on the actual experimental validation of their interesting novel method.

Reviewer #3: The authors addressed all my comments. I am satisfied with these changes and I think that this revision is much improved. I recommend publishing.
